# Text Messaging in the Patient-Centered Medical Home to Improve Glucose Control and Retinopathy Screening

**DOI:** 10.1089/heq.2016.0003

**Published:** 2017-01-01

**Authors:** Janice M. Miller, Ann G. Phalen, Albert Crawford, Anthony Frisby, Barry S. Ziring

**Affiliations:** ^1^ Jefferson College of Nursing, Thomas Jefferson University, Philadelphia, Pennsylvania.; ^2^ College of Population Health, Thomas Jefferson University, Philadelphia, Pennsylvania.; ^3^ Center for Teaching and Learning, Thomas Jefferson University, Philadelphia, Pennsylvania.; ^4^ Department of Medicine, Sidney Kimmel Medical College at Thomas Jefferson University, Philadelphia, Pennsylvania.

**Keywords:** diabetes, diabetes education, text messaging, patient-centered medical home, retinopathy screening, self-care

## Abstract

**Purpose:** To evaluate the effectiveness of a text messaging program (TMP) to improve glucose control, retinopathy screening (RS) rates, and self-care behaviors in patients with uncontrolled type 2 diabetes.

**Methods:** A single-group design with a quasi-systematic random sample (*n*=20) received educational/exhortational text messages on their cellular phones for 3 months. Subjects, 12 of whom identified as a minority ethnicity, were mostly male, aged 27–73 years.

**Results:** Glucose control and RS rates improved significantly. Subjects (>70%) reported changes in self-care behaviors.

**Conclusion:** Leveraging ubiquitous technology, a TMP for patients with limited access to healthcare education, holds promise.

## Introduction

Equity, accessibility, safety, efficiency, timeliness, patient centeredness, and effectiveness are domains identified by the Institute of Medicine (IOM) to define high-quality healthcare. Minority patients and those of low socioeconomic status have difficulty accessing or navigating the health system and thus receive lower quality care.^1^ Type 2 diabetes (T2DM) is a complex disease whose poor control portends life altering, costly complications. T2DM impacts minority populations more frequently than Caucasians.^2^ Preventive screenings and blood glucose control decrease complications or identify them early. Approximately 59% of patients have retinopathy screening (RS) to identify early changes leading to blindness, and 36% have controlled T2DM.^3^ Glucose control is measured using the hemoglobin A1C test (A1C), which measures glucose control over a 3-month period. The target for most patients is ≤7 mg/dL.^4^ Without ongoing education and support, patients often fall short of goals.

Following the example of large-scale public health initiatives, a text messaging program (TMP) for patients with T2DM has the potential to impact patients in short segments.^5^ Leveraging cell phone technology that is ubiquitous in the population affords the opportunity to integrate education in a manner that is equitable, accessible, portable, and efficient. This pilot study evaluated the effectiveness of a TMP in 20 subjects with uncontrolled T2DM to improve A1C, RS rates, and performance of the American Association of Diabetes Educators (AADE) self-care behaviors.^6^ Referred to as Mobile Health, this methodology uses “push” technology, transmitting information without the user initiating the request.^7^ TMPs have demonstrated effectiveness in appointment keeping, vaccine reminders, breastfeeding, weight loss, physical activity improvement, improving nutritional intake, HIV testing, and to support decision-making for parents of critically ill neonates.^8–15^

## Methods

All study procedures were approved by the University's Institutional Review Board. This study utilized a pre- and postsingle-group intervention design in an urban, academic Internal Medicine practice designated as a Level III patient-centered medical home (PCMH) by the National Committee on Quality Assurance (NCQA). Practice data revealed that 55% of patients with diabetes had RS within the last year, a metric close to national averages, but that had not improved despite practice efforts. Quality metrics for glucose control within the practice were similar to national averages of 36%. Adult subjects were eligible to participate if they had T2DM, a most recent A1C reading >7.5 within the prior 3 months, were English speaking, had no cognitive dysfunction, no anemia, and were able to read text messages on their cell phone. A quasi-systematic random sampling methodology was utilized to include patients who had a scheduled appointment with one of each of the 12 full-time providers in the practice. This prevented oversampling of a few providers to better represent the practice's T2DM population. All subjects were recruited over 10 weeks. Informed consent was obtained. Subjects texted the keyword “Diabetes” from their cell phone to an investigator contracted short message service vendor. This anonymously enrolled them in the distribution list. Subjects received an immediate response message welcoming them to the program and confirming their enrollment. Subjects were given the book entitled “Living with Diabetes,” which contains comprehensive self-management information and color pictures.^16^ Medications, A1C readings, and most recent RS dates were extracted from the subjects' Electronic Medical Record (EMR). The categorizations of RS (up to date or not up to date) were defined as a consultation letter of a dilated ophthalmologic examination in the patient's EMR within 12 months before study enrollment.

### Interventions

Subjects received text messages three times throughout the week for 12 consecutive weeks. All enrolled participants received the same message on the same day at the same time. All subjects received the same 36 messages.

Messages were limited by the investigator contracted vendor to 160 characters. The targets of all messages were to improve A1C and RS rates. Information was stated in simple language to be understood by participants with low health literacy. Messages contained phone numbers to procure ophthalmology appointments and tips in self-care formulated from information or standards designated by the American Diabetes Association (ADA) or AADE. Each of the AADE behaviors was addressed in the message content. Numerous messages integrated more than one self-care behavior. Due to heterogeneity of subjects and variety of medications prescribed, messages pertaining to medications were generalized and exhortative. At completion, subjects completed a five-point Likert scale survey of their impressions of the TMP and questions reflecting the self-care behaviors of the AADE.

## Results

### Population characteristics

Subject demographics and baseline characteristics are listed in Table 1. A wide range of subject ages and ethnicities participated. Although relatively equal numbers of men and women were approached for participation, male subjects outnumbered female 3:1. One subject was legally blind due to sarcoidosis, but an application on his cell phone converted text to voice messaging and facilitated his ability to send and receive information. While no subjects actively withdrew from the study, six subjects did not keep follow-up appointments or return the exit survey that was then mailed to them.

**Table 1.** **Subject Demographics (*n*=20)**

Male	15
Female	5
Mean age	51.8
Age range	27–73
Ethnicity	
African American	9
Caucasian	7
Asian	2
Hispanic	1
Other	1
Years since diagnosis	11.3
Mean baseline A1C	9.4%
Range of baseline A1C	7.5–14.2%
Family history of diabetes, *n*	15 unknown/adopted=1
Using insulin, *n*	11
Prior diabetes education, *n*	13

Twelve subjects (60%) completed all pre- and postintervention surveys and laboratory work. Fourteen subjects (70%) completed exit surveys. Paired *t*-tests were performed for pre- and post-A1C readings. Fisher's exact test was utilized to evaluate categorical data of RS. Statistical analysis utilized SAS version 9.2 software.

### Glucose control

Eleven of 14 participants had paired data and demonstrated reductions in A1C. The range of A1C change was from −3.4 to +1.1. Paired *t*-tests of pre- and postintervention A1C (*n*=14) revealed an overall mean reduction of 0.83, from 9.46 to 8.63 mg/dL (*p*=0.033).

Several factors likely impacted A1C results. A 3-month time limitation may have been too soon to see improvement in A1C. Missing test results in a small sample size diminish the external validity of the study. Insulin initiation/intensification in four subjects was likely to have reduced their A1C measurements more substantially than did the concurrent TMP. Yet, 71% of patients reported increased medication adherence, a pivotal component of glucose control. An A1C reduction of 0.83 in 11 participants is not to be discounted. A one-point reduction of A1C reduces the likelihood of long-term complications by 35%.^4^ Pharmacologic agents report a similar degree of A1C reduction after initiation of therapy. A multisession diabetes education program demonstrated a similar degree of reduction to the results from this brief TMP.^17^

### Retinopathy screening

At baseline, six subjects (30%) were considered up to date with RS. At study completion, 11 participants (55%) were up to date (*p*=0.014). Eight subjects (42%) underwent RS during or shortly after the 3 months of the TMP (several who were up to date at study initiation repeated RS).

Fourteen patients were due for RS; five of whom completed RS (36%). The subject group as a whole matched the practice's customary annual RS rate in one-fourth of the time. Perhaps more subjects had screening, but a consultation letter was not sent or scanned into the EMR.

Two confounding variables likely impacted RS rates. The practice location installed a retinal camera 1 month before study initiation; one participant had screening performed with this device. Second, as part of a national study, an ophthalmological institution offered free, impromptu RS in the lobby of the practice's building; three participants had consultation reports from this initiative. The variables, while confounding, removed barriers and facilitated performance of RS in the presence of increased cues for four of the participants. National discussion about changing health behaviors focuses on strategies that make healthy choices easier for the population. Facilitating risk reduction behaviors in the presence of TMP cues may be an optimal strategy.

### Exit surveys and AADE behavior changes

**FIG. 1.**
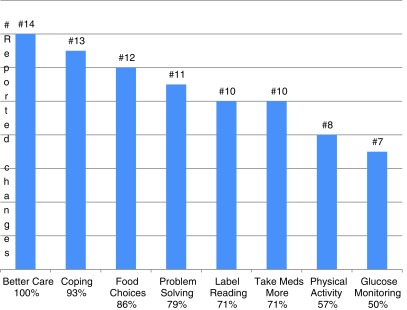
Subject reported self-care behavior improvements. Numbers and percentages of subjects who agreed or strongly agreed that they had made behavior changes by category after the text messaging program: take better care of their diabetes, improved coping skills, made changes to food choices, improved problem solving, increased label reading, taking medications as prescribed, increased physical activity, and self-monitoring blood glucose.

All 14 subjects who completed the exit survey agreed they would recommend the TMP and that it made them want to take better care of their diabetes. No participant felt messages were too frequent. They enjoyed the portability and ability to learn information they “would not seek out in a book or on a computer.” Self-reported changes of AADE behaviors that affect A1C and self-management are listed in Figure 1.

Self-management behaviors are complex and interdependent. Perhaps the combination of messages irrespective of content served as cues to action to engage in self-care behaviors, as patient exit comments included “The messages were frequent reminders to be active in controlling my diabetes” and “Patients still have to take responsibility.”

## Conclusions

Vulnerable populations use text messaging at much higher rates than they use the Internet.^18–21^ Their ability to receive information and cues improves the possibility of engagement in self-management behaviors and improved outcomes. A TMP meets the IOM definition of high-quality care and is consistent with the comprehensive care delivered in a PCMH. Furthermore, a TMP crosses socioeconomic, ethnic, and gender boundaries and permits patients with expensive copays or who do not use the Internet to receive and store portable health information to which they can later refer. Broader and multilingual studies of TMPs are warranted as a population health tool to impact chronic disease and reduce disparities in care.

## Abbreviations Used

AADE: American Association of Diabetes Educators
ADA: American Diabetes Association
EMR: Electronic Medical Record
IOM: Institute of Medicine
NCQA: National Committee on Quality Assurance
PCMH: patient-centered medical home
RS: retinopathy screening
T2DM: type 2 diabetes
TMP: text messaging program
